# Long-chain *n*-3 PUFA in vegetarian women: a metabolic perspective

**DOI:** 10.1017/jns.2017.62

**Published:** 2017-11-23

**Authors:** Graham C. Burdge, Sze-Yen Tan, Christiani Jeyakumar Henry

**Affiliations:** 1 Academic Unit of Human Development and Health, Faculty of Medicine, University of Southampton, Southampton SO16 6YD, UK; 2 Clinical Nutrition Research Centre, Centre for Translational Medicine, Yong Loo Lin School of Medicine, Singapore and Department of Biochemistry, National University of Singapore, Singapore

**Keywords:** EPA, DHA, Vegetarian women, *α*-Linolenic acid, Desaturase, ALNA, α-linolenic acid, FADS, fatty acid desaturase

## Abstract

Vegetarian diets have been associated with health benefits, but paradoxically are low in EPA and DHA which are important for development, particularly of the central nervous system, and for health. Humans have limited capacity for synthesis of EPA and DHA from α-linolenic acid, although this is greater in women than men. Oily fish and, to a lesser extent, dairy foods and meat are the primary sources of EPA and DHA in the diet. Exclusion of these foods from the diet by vegetarians is associated consistently with lower EPA and DHA status in vegetarian women compared with omnivores. The purpose of the present review was to assess the impact of low EPA and DHA status in vegetarian pregnancies on the development and health of children. EPA and DHA status was lower in breast milk and in infants of vegetarian mothers than those born to omnivore mothers, which suggests that in the absence of pre-formed dietary EPA and DHA, synthesis from α-linolenic acid is an important process in determining maternal EPA and DHA status in pregnancy. However, there have been no studies that have investigated the effect of low maternal DHA status in vegetarians on cognitive function in children. It is important to address this gap in knowledge in order to be confident that vegetarian and vegan diets during pregnancy are safe in the context of child development.

## Introduction

Vegetarianism is a widely practised dietary choice that may be adopted for cultural or religious reasons, or out of personal preference, possibly because of perceived health benefits or concerns about animal welfare^(^^1^^)^. The practice of vegetarianism encompasses a range of dietary choices such as the complete exclusion of all meat, fish and dairy produce (veganism), exclusion of meat and fish with inclusion of dairy products and eggs (ovo-lacto-vegetarianism), or exclusion of meat, fish and eggs, but with inclusion of dairy products (lacto-vegetarianism)^(^^1^^)^. For the purpose of the present review, ovo-lacto-vegetarianism will be described as vegetarianism as this is the most common type of vegetarian diet that has been studied previously. There are too few studies that have investigated the effects of other vegetarian dietary practices on *n*-3 PUFA status, with the exception of veganism of which there have been a number of studies and will also be discussed here.

Exclusion of major food groups from the diet may confer a risk of low status or deficiency of nutrients that are found predominately or exclusively in the excluded foods. The *n*-3 PUFA EPA and DHA are obtained primarily from oily fish and, to a lesser extent, from meat and dairy products^(^^2^^)^. Thus excluding these foods from the diet may incur risk of low EPA and DHA status. Lower EPA and DHA intake and status have been associated with adverse effects on cardiovascular health and on inflammatory disease^(^^3^^–^^5^^)^. Furthermore, adequate assimilation of DHA by the developing central nervous system is required for optimal function^(^^6^^)^ and deficits in DHA accumulation have been associated with impaired retinal function in preterm infants^(^^7^^)^ and with altered behaviour in non-human primates^(^^8^^–^^10^^)^. There have been several previous reviews of the impact of vegetarian diets on EPA and DHA status, which concluded that individuals who follow vegetarian diets have lower EPA and DHA status compared with omnivores^(^^11^^–^^15^^)^. Nevertheless, vegetarian diets have been associated with specific health benefits including reduction in CVD risk factors^(^^16^^)^, lower risk of type 2 diabetes mellitus and impaired glucose homeostasis^(^^17^^,^^18^^)^ and the metabolic syndrome^(^^19^^)^, enhanced weight reduction, although effects on maintenance of lower weight are uncertain^(^^20^^)^, reduced risk of cancer^(^^21^^,^^22^^)^, and differential effects of vegetarian and vegan diets on the gut microbiota^(^^23^^)^. Vegetarianism in affluent countries tends to be practised by individuals with higher income and educational attainment, while vegetarianism in less wealthy counties is associated primarily with poverty, and religious or cultural practice^(^^24^^)^. Thus, it has been suggested that at least part of the beneficial effects of vegetarianism on health may be attributed to lifestyle rather than dietary practice *per se*^(^^24^^)^.

DHA status has been shown to be higher in women than men^(^^25^^)^, partly because women have been shown to be able to convert the essential fatty acid α-linolenic acid (ALNA) to DHA^(^^26^^)^, while such capacity in men is negligible^(^^27^^)^. It is thought that such metabolic capacity in women may facilitate the supply of DHA to their offspring. Because DHA is important for neurological development, low DHA status in non-pregnant and pregnant vegetarian women may adversely affect the development of children born to vegetarian mothers. The purpose of the present review was to compare the impact of vegetarian diets on EPA and DHA status in non-pregnant and pregnant women, and to assess whether maternal vegetarian diet affects the development of their children.

### *n*-3 Fatty acids: dietary sources

*n*-3 Fatty acids are long-chain PUFA that are characterised by the presence of a double bond on the third carbon from the methyl end of the hydrocarbon chain. They can be grouped into the 18-carbon fatty acids ALNA and stearidonic acid (18 : 4*n*-3) that are present in vegetable oils, and those with chains of 20 or more carbons found in animal products, especially oily fish: 20 : 4*n*-3, EPA, DPA*n*-3 (22 : 5*n*-3) and DHA. ALNA is synthesised in plants from oleic acid (18 : 1*n*-9) by sequential reactions that are catalysed by ∆12 and ∆15 desaturases^(^^28^^)^. Plants of the Boraginaceae are able to synthesise stearidonic acid by ∆6 desaturase activity, and may contain between 5 and 10 % of this PUFA^(^^29^^)^. Humans and other mammals are unable to synthesise ALNA because they lack ∆12 and ∆15 desaturases and consequently this fatty acid is regarded as essential in the diet^(^^30^^)^. Poor ALNA intake, primarily in patients receiving total parenteral nutrition, has been linked to specific deficiency symptoms^(^^31^^)^. Therefore, in order to maintain adequate EPA and DHA status, humans and other mammals are dependent upon either consuming these fatty acids pre-formed in the diet from animal-derived foods^(^^2^^)^ and/or on synthesis from ALNA.

### Synthesis of EPA and DHA in humans

The consensus pathway for conversion of ALNA to longer-chain *n*-3 PUFA in mammals was elucidated by Voss & Sprecher^(^^32^^)^ in rat liver and involves sequential desaturation and carbon chain elongation reactions. The initial rate-limiting reaction introduces a double bond into ALNA at the ∆6 position and is catalysed by ∆6 desaturase to produce 18 : 4*n*-3, which is then converted to 20 : 4*n*-3 by the addition of two carbons by elongase 5 activity. Desaturation of 20 : 4*n*-3 to form EPA is catalysed by ∆5 desaturase. Chain elongation of EPA to DPA*n*-3 is catalysed by elongase 2 or 5, and then DPA*n*-3 can be elongated to 24 : 5*n*-3 by elongase 2 activity. Desaturation of 24 : 5*n*-3 at the ∆6 position by ∆6 desaturase produces 24 : 6*n*-6. Then 24 : 6*n*-3 is translocated from the endoplasmic reticulum to peroxisomes where two carbons are removed to form DHA. It has been suggested that the reactions downstream of DPA*n*-3 may regulate DHA synthesis independent of the synthesis of DPA*n*-3^(^^32^^)^.

Although this pathway is active in rodents, and possibly other species, humans appear to have limited capacity for the synthesis of EPA, DPA*n*-3 and, in particular, DHA. Dietary supplementation studies in men alone or men plus postmenopausal women show an increment in EPA in blood lipids that was related to the level of ALNA intake, but no increase in DHA^(^^33^^)^. These findings are supported by the results of studies in which men who consumed ALNA labelled with a stable isotope tracer showed synthesis of EPA, DPA*n*-3, but not DHA^(^^27^^)^. In addition, James *et al*.^(^^34^^)^ conducted a study to attempt to overcome the point of constraint in ALNA conversion. Men and postmenopausal women consumed either ALNA, 18 : 4*n*-3, which would be expected to overcome any constraint in desaturation of ALNA by ∆6 desaturase, or EPA, which would be expected to overcome any constraint in conversion due to ∆6 desaturase, ∆5 desaturase and elongase 5. All three interventions induced an increase in EPA and DPA*n*-3 status, but not in DHA^(^^34^^)^. In some dietary supplementation studies, feeding ALNA, 18 : 4*n*-3 or EPA was associated with a decrease in DHA status^(^^33^^)^. This may reflect retro-conversion of DHA to shorter-chain, less unsaturated *n*-3 PUFA in the absence of dietary input of pre-formed DHA^(^^35^^)^. Based on these findings, it has been concluded that capacity for conversion of ALNA to EPA is limited and to DHA is severely constrained in men and post-menopausal women^(^^26^^)^. However, conversion of stable isotope-labelled ALNA to EPA and DHA was markedly greater in young women than in men^(^^36^^)^. This apparent sex difference is supported by a meta-analysis of fifty-one studies which showed that typically women have a 20 % higher proportion of DHA in blood lipids than men^(^^25^^)^. Higher DHA status in females compared with males has also been reported in rodents and wild birds (*Parus major*)^(^^37^^)^, which suggests that this sex difference may either be conserved or the result of convergent evolution. If so, this implies that higher DHA status and capacity for synthesis is of biological importance in females. Furthermore, female sex hormones, specifically 17-α-ethynyl oestradiol and progesterone have been shown to increase DHA synthesis^(^^36^^)^ or are associated with higher DHA status^(^^38^^,^^39^^)^. A recent study in HepG2 cells and human primary hepatocytes showed that progesterone, but not 17-α-ethynyl oestradiol or testosterone, increased EPA, DPA*n*-3 and DHA synthesis, up-regulated the mRNA expression of fatty acid desaturase 1 (*FADS1*) and *FADS2*, which encode ∆5 or ∆6 desaturases, respectively, and elongation of very long-chain fatty acids 2 (*ELOVL2*) and *ELOVL5*, which encode elongases 2 and 5, respectively^(^^40^^)^. Furthermore, progesterone induces reduction in the DNA methylation of specific loci in the 5-regulatory region of its gene *FADS2*, which suggests that the difference in DHA synthesis and status between men and women may involve differential epigenetic regulation^(^^40^^)^. The biological significance of EPA and DHA synthesis in women has yet to be demonstrated directly.

### PUFA metabolism in women

DHA status in women and rodents has been shown to increase during pregnancy^(^^41^^–^^43^^)^ and there is selective DHA enrichment in maternal hepatic and/or plasma phospholipids^(^^43^^–^^46^^)^ by a mechanism that involves altered acyl remodelling of phosphatidylcholine synthesised *de novo*^(^^45^^)^. Furthermore, the concentration of DHA in umbilical cord blood has been shown to be greater than that in the maternal circulation in humans^(^^44^^,^^47^^)^ and guinea pigs^(^^48^^)^, although the mechanism underlying the placental biomagnification of PUFA has not been characterised. Together, these processes may facilitate the supply of DHA from mother to offspring and buffer variation in supply of pre-formed DHA from the mother.

## EPA and DHA intakes in vegetarian and vegan women

A number of governments and other organisations have published recommendations for combined intakes of EPA and DHA which range between 250^(^^49^^)^ and 1000 mg/d^(^^50^^)^, primarily to promote cardiovascular health. Relatively few of the studies that investigated the effect of vegetarian diets on EPA and DHA status in women have also reported the dietary intakes of these fatty acids. Welch *et al*.^(^^51^^)^ reported that UK vegetarian women consumed approximately 10 mg/d EPA and <1 mg/d DHA, and that vegan women consumed 20 mg/d EPA and no detectable DHA compared with fish-eating omnivore women (EPA 110 mg/d; DHA 150 mg/d). Lakin *et al*.^(^^52^^)^ estimated EPA and DHA intake of 17 mg/d in Scottish vegetarian mothers (*n* 4) who had recently given birth compared with omnivore mothers (316 mg/d). These findings suggest that vegetarian and vegan women do not meet recommended EPA and DHA intakes and that their consumption of these fatty acids is markedly below that of their omnivore counterparts.

Only one study has reported the dietary sources of EPA and DHA in vegetarians and vegans^(^^51^^)^. Fat spreads accounted for 59 % of EPA consumed by UK vegetarian women while a further 26 % was provided by dairy foods and a further 6 % by eggs and 6 % from cereals. DHA was obtained from eggs (88 %) and from soups and sauces (12 %). In vegan women, EPA was obtained primarily from soups and sauces (76 %) and from spreading fats (23 %). DHA intake was too low to be measured in vegan women. Eggs and butter contain EPA and DHA. However, it is somewhat surprising that spreads and soups consumed by vegans, who do not consume dairy products or eggs, contained EPA since it is found only in foods derived from meat or oily fish. Unfortunately, the authors did not disclose which foods provided EPA in the diets of the vegan women.

ALNA intake in UK vegetarian women of 0·97 g/d was similar to fish-eating omnivores (0·99 g/d), although ALNA consumption in vegans (0·86 g/d) was lower than both of these groups^(^^51^^)^. ALNA intake in Scottish vegetarian women (1·5 g/d) was also similar to that of omnivores (1·2 g/d)^(^^52^^)^. Linoleic acid intake was also similar between vegetarian (9·6 g/d) and omnivore women and the 18 : 2*n*-6/ALNA ratio did not differ significantly between vegetarians (7·0 g/d) and omnivores (7·9 g/d)^(^^52^^)^. Although the evidence is limited, these findings suggest that, perhaps counter-intuitively, dietary intake of plant-derived essential fatty acids was not greater in vegetarians. This has implications for the influence of vegetarian diets on the capacity of vegetarian women to convert ALNA to longer-chain *n*-3 PUFA compared with omnivores. Since their intake of ALNA substrate and the relative intakes of essential fatty acids which compete for Δ6 desaturase were similar between the dietary groups, the capacity for synthesis of longer-chain *n*-3 PUFA may be expected to be comparable, except omnivores would be expected to have lower capacity for conversion of ALNA due to product inhibition by pre-formed dietary EPA and DHA.

Mead acid (20 : 3*n*-9) and DPA*n*-6 are markers of low essential fatty acid and DHA intakes, respectively^(^^8^^,^^53^^)^. The concentration of 20 : 3*n*-9 did not differ significantly between ovo-lacto-vegetarians and omnivores, although only one study to date has reported the level of this fatty acid^(^^54^^)^. However, the concentration of 22 : 5*n*-6 has been shown to be increased in ovo-lacto-vegetarians or vegans in one study^(^^55^^)^, but not others^(^^52^^,^^56^^)^. This suggests that at least some groups of vegetarians are at risk of DHA deficiency, although it is unclear why this cohort^(^^55^^)^ had an elevated 22 : 5*n*-6 concentration compared with participants in other studies.

## EPA and DHA status in vegetarian and vegan women

The findings of studies that compared EPA and DHA status in vegetarian women with individuals with other dietary practices are summarised in Table 1. These studies have been carried out in geographically diverse locations, with differing classifications of diet, and using different lipids (plasma or serum phospholipids, total serum fatty acids or erythrocyte membrane phospholipids) as the primary outcomes. Nevertheless, despite these potential confounding influences, consistent significant differences between vegetarians, vegans and those following other dietary practices have been reported.

**Table 1.** Summary of studies that have compared *n*-3 PUFA status in omnivore and vegetarian (Mean values and standard deviations)

				ALNA		EPA		DPA*n*-3		DHA	
Study	Marker	Subjects and nationality	*n*	Mean	sd	Mean	sd	Mean	sd	Mean	sd
Li *et al.*^(^^57^^)^	Serum phospholipids (% total fatty acids)	Australian									
		Omnivores	24	0·23	0·10	0·95	0·46	0·85	0·22	3·51	0·64
		Vegetarian	50	0·24	0·10	0·60	0·29	0·91	0·25	2·80	0·86
		Difference between groups (*P*)		NS		0·003		NS		<0·0001	
Welch *et al.*^(^^51^^)^	Plasma phospholipids (μmol/l)	UK									
		Fish eaters	1891	12·4	6·1	64·7	43·4	71·8	29·6	271·2	113·1
		Meat eaters	309	13·1	7·3	57·1	38·4	74·7	34·2	241·3	109·6
		Vegetarians	51	12·3	4·8	55·1	52·5	75·0	32·2	223·5	137·8
		Vegans	5	13·7	8·1	50·0	29·4	90·6	54·0	286·4	211·7
		Difference between groups (ANOVA *P*)		NS		<0·001		NS		<0·001	
Melchert *et al.*^(^^58^^)^	Total serum lipids (% total serum fatty acids)	Germany									
		Female non-vegetarians	37	1·10	0·35	ND		ND		2·36	0·59
		Female vegetarians	38	1·35	0·81	ND		ND		1·42	0·47
		Difference between groups (*P*)		< 0·01						<0·001	
Reddy *et al*.^(^^59^^)^	Plasma phospholipids (% total fatty acids)*	UK									
		Caucasian omnivore	24	ND		0·97		ND		2·26	
		South Asian	24	ND		0·36		ND		1·20	
		Difference between groups (*P*)				<0·001				<0·001	

ALNA, α-linolenic acid; ND, not determined.

* Mean values.

Li *et al*.^(^^57^^)^ compared Australian vegetarian women (subjects who did not consume red meat and ate fish or chicken less than once per week for at least 6 months) and omnivores (defined as subjects who ate red meat at least five times per week); both groups aged 20 to 45 years. There were no significant differences in the proportions of ALNA or DPA*n*-3 in plasma phospholipids between vegetarians and omnivores (Table 1). However, there were lower proportions of EPA (37 %) and DHA (20 %) in plasma phospholipids in vegetarians compared with omnivores. Melchert *et al*.^(^^58^^)^ found that, in German women, the proportion of ALNA in total serum fatty acids was 23 % higher and the proportion of DHA was 40 % lower in vegetarians than in omnivores (Table 1). The proportions of EPA and DPA*n*-3 were not reported. Moreover, Reddy *et al*.^(^^59^^)^ showed that for South Asian vegetarian women living in the UK, EPA and DHA were not detected in their diets and that the proportion of EPA in plasma phospholipids was 63 % and DHA 47 % lower than in Caucasian omnivorous women (Table 1). The mean proportions of *n*-3 PUFA in plasma phospholipids across studies that used comparable measurements of fatty acid status^(^^57^^–^^59^^)^ were, for omnivores, ALNA 0·67 %, EPA 0·96 %, DPA*n*-3 0·85 % and DHA 3·0 %, and, for vegetarians, ALNA 0·80 %, EPA 0·48 %, DPA*n*-3 0·90 % and DHA 1·8 % (Table 1). Vegetarians and vegans consumed less EPA and DHA, and had lower concentrations of these fatty acids in blood lipids than omnivores. However, in marked contrast to other studies, Welch *et al*.^(^^51^^)^ showed that despite a 25 % lower intake of EPA + DPA*n*-3 + DHA compared with fish-eating omnivores, vegan women in a cohort in the UK had 6 % more EPA + DPA*n*-3 + DHA in their plasma phospholipids than omnivore women (Table 1). One interpretation is that desaturation and elongation of ALNA to DHA was an important source of DHA in vegetarian and vegan women, although EPA and DHA biosynthesis appears to be insufficient to compensate completely for low intakes of these fatty acids. Furthermore, it cannot be concluded from these findings that low intakes of pre-formed EPA and DHA induce increased synthesis from ALNA and some authors have argued against the suggestion of up-regulation of EPA and DHA synthesis in vegetarians^(^^60^^)^.

## Effect of maternal vegetarian and vegan diets on EPA and DHA status in mothers and infants

Adequate provision of DHA during early life is critical for development, particularly of the central nervous system^(^^6^^)^. Since DHA status and intakes are low among vegetarian and vegan women, it is possible that they may not be able to provide sufficient DHA to the fetus or infant to ensure adequate development.

To date, there have been few studies that have investigated specifically the effect of a maternal vegetarian or vegan diet on EPA and DHA status in infants. DHA concentration in umbilical cord plasma phospholipids was 32 % lower in UK Hindu vegetarians (*n* 27) compared with matched Caucasian omnivores^(^^54^^)^. These findings suggest that the supply of DHA from mother to fetus may be lower in vegetarians than omnivores. The same authors also showed that EPA and DHA intakes in lactating and non-lactating Hindu and Caucasian ovo-lacto-vegetarians were almost undetectable, and EPA and DHA intakes were too low to be measured in vegans. In contrast, omnivores consumed approximately 80 mg EPA/d and between 40 and 100 mg DHA/d^(^^54^^)^. Others have also found EPA and DHA intakes to be below the level of detection in pregnant vegetarian women^(^^61^^)^.

The proportion of DHA in breast milk was 62 % lower in vegans (*n* 19) and 19 % lower in Hindu vegetarians (*n* 5) compared with omnivores (*n* 21)^(^^54^^)^, presumably reflecting lower intake. Furthermore, the proportion of DHA in erythrocyte phospholipids in 14-week-old breast-fed infants of vegan mothers (*n* 3) was 69 % lower than that of omnivore mothers (*n* 6), and was 49 % lower than in infants who were fed cows’ milk formula (*n* 12)^(^^54^^)^, although it is difficult to be confident that this small number of subjects is representative of the wider vegetarian population. However, the same group also reported 80 % lower EPA concentration in breast milk of Caucasian vegans compared with that of omnivores^(^^55^^)^. Mead acid represented 0·2 % total fatty acids in erythrocytes from infants breast-fed by omnivore women (*n* 3), but only a trace amount was detected in infants of omnivores (*n* 6)^(^^54^^)^. This may reflect the lower concentrations of ALNA (47 %) and linoleic acid (78 %) in breast milk of omnivores compared with that of vegan women. Together, the findings of these studies suggest that despite women being able to convert ALNA to EPA and DHA, and metabolic adaptations that increase maternal DHA concentration during pregnancy, vegan and vegetarian women do not appear to able to compensate metabolically for low intakes of pre-formed EPA and DHA during pregnancy.

Lakin *et al*.^(^^52^^)^ found that in Scottish vegetarian mothers (*n* 4) the proportion of EPA was 30 % and of DHA 15 % lower in total placental lipids compared with omnivore (*n* 10) mothers. In addition, EPA was undetectable and DHA 43 % lower in umbilical cord tissues from vegetarian pregnancies compared with omnivores^(^^52^^)^. Reddy *et al*.^(^^59^^)^ have shown that the proportions of EPA and DHA in erythrocyte phospholipids of 14-week-old infants breast-fed by vegan mothers (*n* 3) were lower than in infants breast-fed by omnivore mothers (EPA 71 %; DHA 40 %) or born to omnivore mothers and fed cows’ milk formula (EPA 83 %; DHA 49 %). These findings were confirmed by Sanders *et al*.^(^^55^^)^ who showed that the proportions of EPA and DHA in erythrocytes of infants of at least 3 months of age breast-fed by vegan mothers (*n* 3) were lower (EPA 71 %; DHA 69 %) than those of infants breast-fed by omnivore mothers (*n* 6). Together these data suggest that adaptions to maternal PUFA metabolism that increase DHA status during pregnancy^(^^43^^)^, the capacity of women to convert ALNA to longer-chain *n*-3 PUFA^(^^36^^)^ and biomagnification of DHA by the placenta^(^^44^^,^^47^^)^ were not able to compensate for the absence of pre-formed EPA and DHA either before birth and during breast-feeding. Since low intakes of DHA during infancy have been associated with a deficit in the accumulation of DHA in brain phospholipids in term infants^(^^62^^,^^63^^)^, these findings suggest a potential concern about a negative impact of maternal vegetarian, in particular the vegan diet, on the development of the central nervous system of infants.

### Vegetarian diets, DHA and child development

To date, there have not been any studies to determine whether low DHA status in infants of vegetarian or vegan mothers has any significant adverse effect on their development^(^^64^^)^ and it has been argued that lifelong DHA insufficiency in vegans may increase risk of cognitive decline^(^^65^^)^. However, some insights may be gained from studies of the effect of low maternal fish intake on infant development. One study has investigated the relationship between maternal fish intake and *n*-3 long-chain PUFA status in pregnant Indian women and the birth weight of their infants^(^^66^^)^. Women who did not consume fish during their third trimester had an adjusted OR of 2·49 (95 % CI 1·16, 5·36; *P* = 0·019) for the risk of delivering a low-birth-weight infant and low DHA during pregnancy has been implicated in an increased risk of preterm delivery^(^^67^^)^. Others have also shown that low consumption of seafood is a risk factor for preterm delivery^(^^68^^)^. In addition, low umbilical cord plasma DHA concentration has been associated with impaired mental and psychomotor development at the age of 6 months^(^^69^^)^. However, without appropriate assessment of neurological development in children of vegetarian mothers and other potential confounding factors such as socio-economic status, it is not possible to conclude whether low DHA intake and status affect the neurological development of children negatively.

The potential risk of impaired development as a consequence of low DHA supply from mother to fetus is of particular importance to populations that consume predominately a vegetarian diet. For example, the diet of the Indian population is based mainly on cereals with some use of ALNA-rich oils^(^^70^^)^. The lack of pre-formed EPA and DHA in the diet may, therefore, have a negative impact on development and wellbeing at the population level, although at present there is no evidence to support or refute this suggestion. A recent study has examined allele frequency of the FADS2 rs66698963 polymorphism in 234 Indian vegetarians and 311 Americans^(^^71^^)^. This 22-bp insertion/deletion mutation has been associated with basal FADS1 expression, and with up-regulation of fatty acid desaturase genes *FADS1* and *FADS2*^(^^72^^)^. Kothapalli *et al.*^(^^71^^)^ found that the insertion allele, which was associated with increased expression of FADS1 and FADS2, was found in 68 % Indians, compared with 18 % of Americans. Thus, this allele seems to have evolved in populations that predominantly consumed a plant-based diet for millennia and may enable them to convert *n*-3 and *n*-6 fatty acids to long-chain PUFA more efficiently than in populations that habitually consume omnivorous diets (although the study only focused on *n*-6 PUFA).

### Dietary supplementation with *n*-3 PUFA in vegetarians

Dietary supplementation with EPA and DHA to improve status presents a challenge in non-fish-eating vegetarians and vegans. TAG extracted from marine algae represent one potential source of EPA and DHA that is consistent with vegetarian diets^(^^73^^)^. Unsurprisingly, consuming algal oil supplements increased the concentration of DHA in blood from vegetarian men and/or women^(^^35^^,^^65^^,^^74^^–^^76^^)^, although one study showed an increase in total and LDL-cholesterol concentration following the intervention^(^^75^^)^. One study has investigated the effect of a food-based dietary intervention on EPA and DHA status in North American lacto-ovo-vegetarian men and women^(^^77^^)^. Subjects consumed either DHA-enriched eggs (DHA 500 mg, EPA 40 mg, ALNA 1 g/yolk) or standard eggs (DHA 110 mg, EPA negligible, ALNA 0·15 g). The DHA-enriched eggs increased dietary DHA intake by 4·5-fold compared with the standard egg diets. Consumption of enriched eggs increased the proportion of DHA, but not EPA, in erythrocyte phospholipids. Another group in the same study consumed walnuts (2·95 g ALNA) which provided 3-fold more ALNA than the standard egg diet. Consuming walnuts was associated with a significant decrease in the proportion of DHA in erythrocyte phospholipids, but no change in the proportion of EPA in contrast to other studies in which ALNA intake was increased^(^^33^^)^. These studies show that specific dietary strategies that are potentially acceptable by vegetarians or vegans are effective in increasing EPA and DHA status.

## Conclusions

Studies published to date show, with few exceptions, that EPA and DHA intakes and status in vegetarians and vegans are lower than in omnivores. Synthesis of EPA and DHA may be an important source of these fatty acids in vegetarians and, in particular, vegan women and there is no evidence of metabolic compensation for low intakes of EPA and DHA. In addition, infants born to vegan mothers have lower EPA and DHA status than those born to omnivore mothers. However, the interpretation of these is constrained by the relatively few studies of pregnant and non-pregnant vegetarian women and their offspring. Furthermore, the studies of EPA and DHA status in infants have included very small numbers of subjects and so there is a risk of confounding and of limited extrapolation to the wider population. Furthermore, there have not been any studies of the effect of low DHA status due to maternal vegetarian or vegan diets on cognitive function in children. This represents an important knowledge gap which needs to be addressed in order to be confident that vegetarian and vegan diets during pregnancy are safe in the context of child development and to be able to make appropriate nutritional recommendations.
